# Supplementary material for: Interphase chromosomes of the Aedes aegypti mosquito are liquid crystalline and can sense mechanical cues
Source: Nat Commun. 2023 Jan 19;14:326. doi: 10.1038/s41467-023-35909-2 (PMC9852290; doi:10.1038/s41467-023-35909-2)
Supplement: Supplementary file 1 — Supplementary Information [file 41467_2023_35909_MOESM1_ESM.pdf]

Supplementary Information: Interphase chromosomes of the Aedes aegypti mosquito are liquid crystalline and can sense mechanical cues

Vinícius G. Contessoto,[1,2] Olga Dudchenko,[1,3] Erez Lieberman Aiden,[1,3] Peter G. Wolynes,[1,4,5,6] José N. Onuchic,[1,4,5,6] and Michele Di Pierro[7,8]

[1] *Center for Theoretical Biological Physics, Rice University, Houston, TX, USA*

[2] *Instituto de Biociências, Letras e Ciências Exatas, UNESP - Univ. Estadual Paulista, Departamento de Física, São José do Rio Preto, SP, Brazil*

[3] *The Center for Genome Architecture, Department of Molecular and Human Genetics, Baylor College of Medicine, Houston, TX, USA*

[4] *Department of Physics & Astronomy, Rice University, Houston, TX, USA*

[5] *Department of Chemistry, Rice University, Houston, TX, USA*

[6] *Department of Biosciences, Rice University, Houston, TX, USA*

[7] *Department of Physics, Northeastern University, Boston, MA, USA*

[8] *Center for Theoretical Biological Physics, Northeastern University, Boston, MA, USA*

E-mail: vinicius.contessoto@rice.edu; jonuchic@rice.edu; dipierro@northeastern.edu

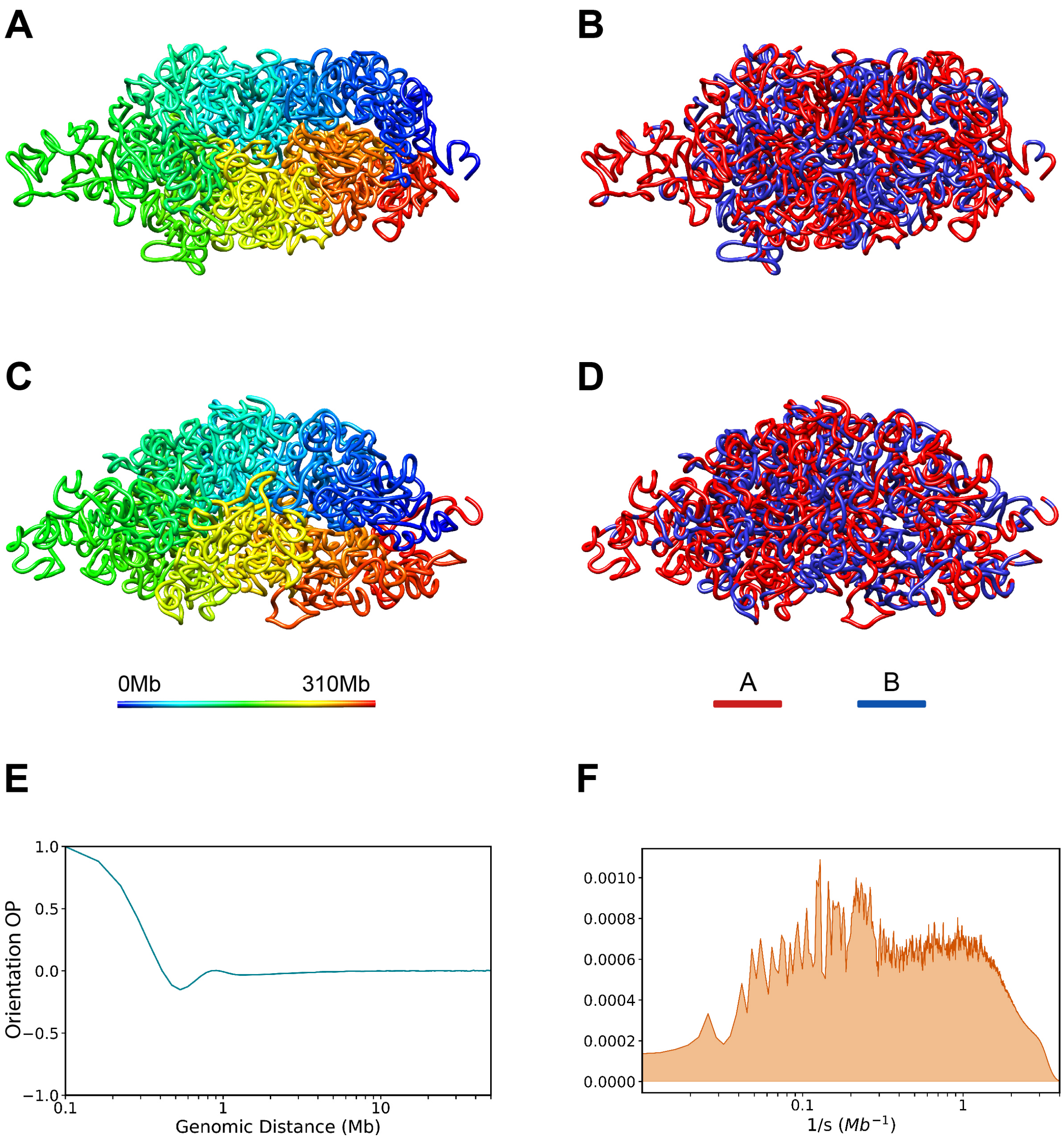

Supplementary Fig. 1: Representative 3D structures of chromosome 1 from *Aedes aegypti* modeled at the nominal information theoretic temperature ($T = 1.0$).[1] A and C represent the structures colored based on the sequential genomic position of each locus. The range goes from blue to red, start and end of the polymer chain, respectively. B and D show the 3D structures painted using the 1D A/B types information extracted from the first component of the eigenvector. E and F - The orientation order parameter (O_{OP}) as a function of the genomic distance and its Fourier transform, respectively. O_{OP} is an average calculated over the ensemble of 3D structures at T=1.0 (see O_{OP} calculation below)

.

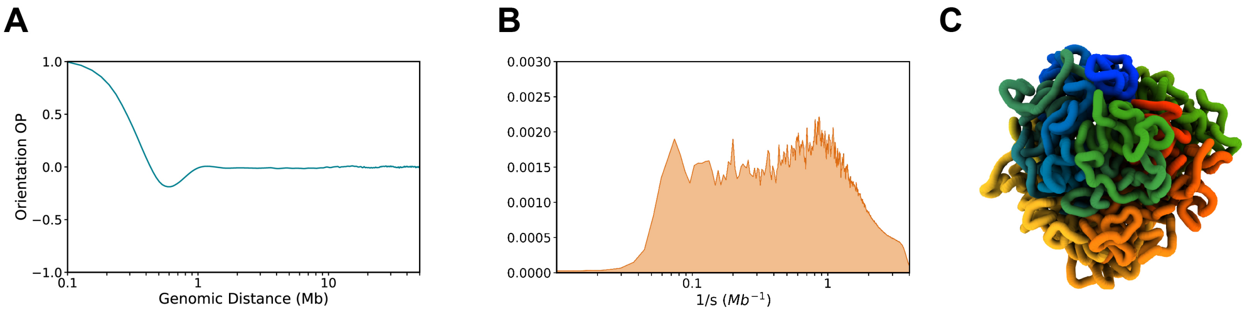

Supplementary Fig. 2: A and B - The orientation order parameter (O_{OP}) as a function of the genomic distance and its Fourier transform for the Human chromosome 10 at 100kb resolution, respectively. O_{OP} is an average calculated over the ensemble of 3D structures at T=1.0 (see O_{OP} calculation below). C - Representative Representative 3D structures of chromosome 10 at 100kb from the Human cell line GM12878.

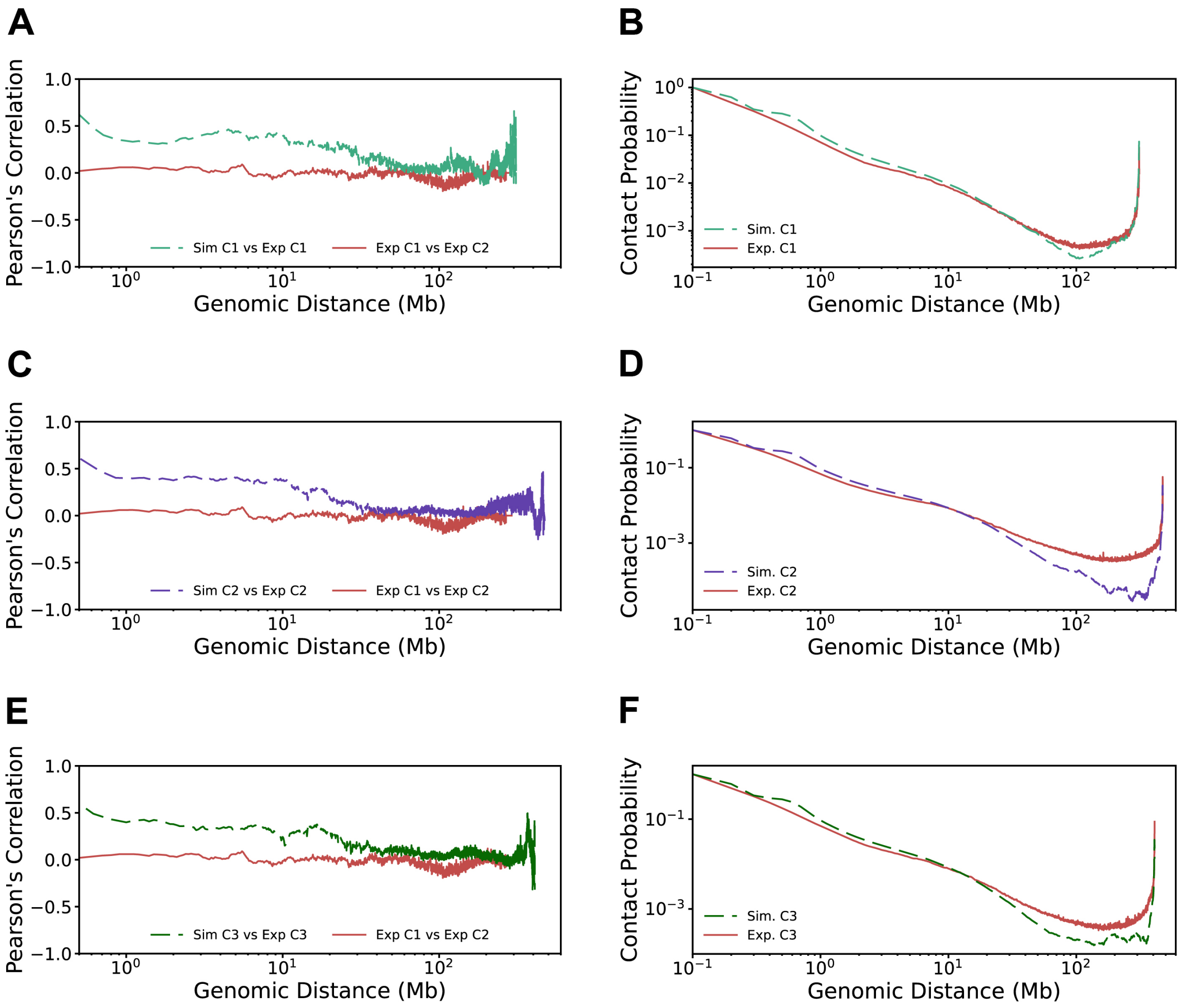

Supplementary Fig. 3: Pearson's correlation and contact probability as a function of the genomic distance.[2–5] The curves were obtained for each chromosome extracted from the *Aedes aegypti* full nucleus simulation. The red curves on A, C, and E correspond to a correlation baseline (comparing chromosomes 1 and 2 from experimental Hi-C maps) that highlights the modeling correlation (comparing the simulated and experimental Hi-C maps) that is higher when compared to a baseline as a function of genomic distance. The red curves on B, D, and F correspond to a correlation baseline (comparing chromosomes 1 and 2 from experimental Hi-C maps). The modeling correlation (comparing the simulated and experimental Hi-C maps) is significantly higher than the red baseline curve as a function of genomic distance. The labels 1M and 1P in the main text are used for distinguishing between the two copies of the same chromosome when simulating the full nucleus. C1 is presented here as data obtained for the chromosome without distinguishing between homologous chromosomes.

Supplementary Discussion

Comparison Between Human and Mosquito Genomes Using ACA Score

In our recent work,[6] we performed in situ Hi-C on 24 species, and the architectural features observed in those maps can be divided into two groups, type-I and type-II. Type-I group (that includes the *Aedes aegypti*) incorporates Rabl-like configurations such as centromere clustering, telomere clustering, and telomere-to-centromere axis. Type-II group (human and other mammalians) includes the genome organization related to chromosome territories. These architectural features are identified using a scoring parameter called ACA (Aggregate Chromosome Analysis). ACA aggregates the signal from all intra- and inter-chromosomal contacts, and the scores are defined as observed-to-expected ratios (see Supplementary Information ref[6] for details). The ACA chromosome territory score $S(ct)$ for the *Aedes aegypti* is 1.109 which is low when compared to 11.150 from the Human genome or 6.179 from the Tammar wallaby chromosomes (mammalian). These ACA values defined the Human and Tammar wallaby genomes to belong to the type-II group which forms territories. On the other hand, the low value of $S(ct)$ includes the mosquito in group type-I (see Supplementary Tables S3 and S8 of ref[6]).

In addition to this ACA score, we compare the contact probability as a function of the genomic distance for chromosome 1 from *Aedes aegypti* and human cell line GM12878[7] (Supplementary Fig. 4). The curves have similar decay until the region around 2 Mb where the mosquito genome (solid red curve) shows higher probabilities than the human (dashed blue line) until it reaches the values close to 11 Mb. The difference in the decay in this length scale is related to higher compaction of the Aedes genome. On the other hand, for genomic separation longer than 11 Mb, the human chromosome shows a higher contact frequency in comparison to the mosquito which suggests the formation of long-range interaction and the formation of territories.

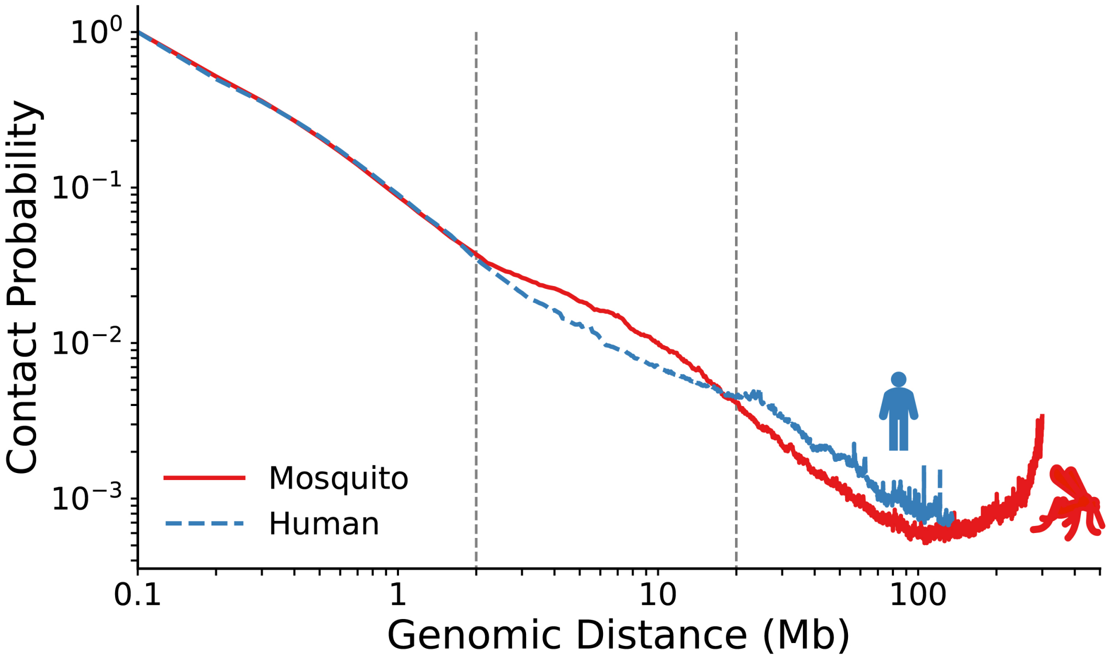

Supplementary Fig. 4: Contact probability as a function of genomic distance obtained from experiments. The solid red curve represents the data extracted from the Hi-C maps for chromosome 1 of *Aedes aegypti*.[8,9] The dashed blue line represents the data obtained from the human chromosome 1 from the GM12878 cell line Hi-C map.[7]

Comparison Between Different Matrix Balancing Methods

The Hi-C map used in the model training was balanced using the KR - Knight-Ruiz[10] algorithm that is implemented in Juicer software package.[11] In order to address the possible artifacts generated by the normalization, we calculated the contact frequency as a function of the genomic distance for the *Aedes aegypti* chromosome 1 using three different normalization methods available in Juicer and raw data (un-normalized). Supplementary Fig. 5 presents the scaling curves that do not present significant variations when comparing different normalization methods. We can then assume that there is no over- or underestimation of short- or long-range contacts due to matrix balancing.

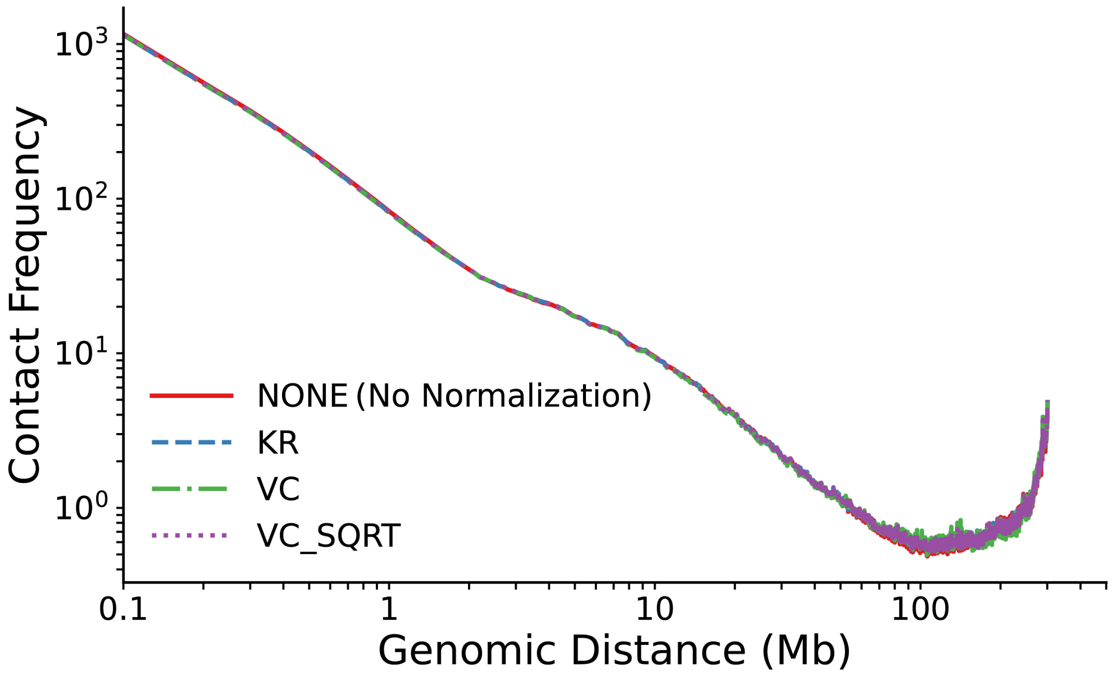

Supplementary Fig. 5: Contact probability as a function of the genomic distance of *Aedes aegypti* chromosome 1 extracted using different normalizations.

Correlation Between A/B Compartments and High/Low ATAC-seq Intensity Values

We use the first component of the eigenvectors extracted from the correlation matrix of the experimental Hi-C map to determine the A/B compartments. The ATAC-seq signals are divided into two groups based on the signal intensity, High and Low. The group denominated High contains signal values above the 95th percentile (colored in green - Supplementary Fig. 6A). On the other hand, the group Low corresponds to signal values below the 5th percentile (colored in gray- Supplementary Fig. 6A). There are a total of 156 elements in each percentile cut. From the High group, 100 loci are identified belonging to the A compartment (red area in Supplementary Fig. 6B) which corresponds to 64% agreement of a locus being classified as A type and having a high signal value of the ATAC-seq track. In addition, there are 91 loci identified as B compartments (blue area in Supplementary Fig. 6B) that give 59% hits.

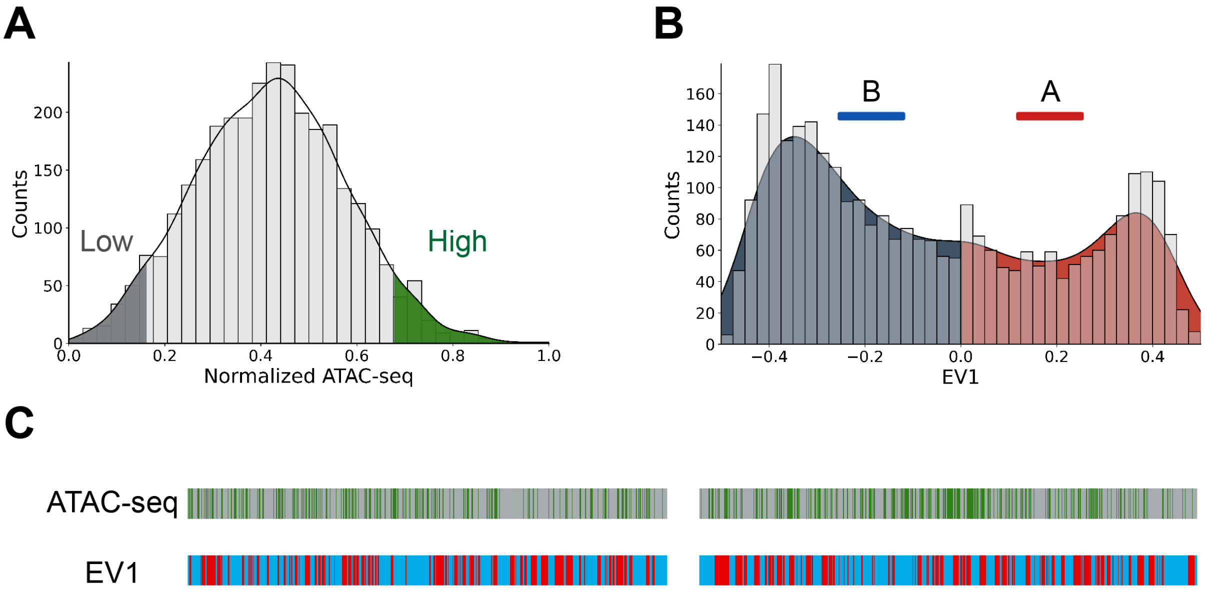

Supplementary Fig. 6: A - ATAC-seq normalized (min-max normalization) signal distribution. High (green) and Low (gray) correspond to 95th and 5th percentile, respectively. B - Distribution of the first component of the eigenvector of the Pearson correlation matrix extracted from the Hi-C map of chromosome 1 of the *Aedes aegypti*. Red and blue correspond to A and B chromatin types. C - Color diagram of the EV1 and ATAC-seq signal for each locus chromosome 1 at 100 kb resolution.

Supplementary Table 1: Kolmogorov-Smirnov statistic two-sided test (KS-value) comparing the High and Low ATAC-seq value distributions. The test computes the null hypothesis that two independent data sets are obtained from the same continuous distribution.[12] The distributions are presented in Figure 3D of the main text.

**Chromosomes**	**KS-value**
1P and 1M	0.26
2P and 2M	0.52
3P and 3M	0.27
Whole Genome	0.58

Supplementary Methods

Homopolymer Model

The homopolymer potential employed here describes a generic bead-spring polymer in which each bead represents a genomic segment of 100 kb in sequence. The potential energy $U_{HP}(\vec{r})$ describes a spatially self-avoiding polymer and serves as a support for the features added by using the maximum entropy principle.[1–3,5] This potential consists of the following four terms, U_{FENE}, U_{Angle}, U_{hc} and, U_{sc}:

$$\begin{aligned} U_{HP}(\vec{r}) &= \sum_{i\in\{\text{Loci}\}} U_{\text{FENE}}(\vec{r}_{i,i+1}) + \sum_{i\in\{\text{Loci}\}} U_{hc}(\vec{r}_{i,i+1}) + \sum_{i\in\{\text{Angles}\}} U_{\text{Angle}}(\theta_i) \\ &+ \sum_{\substack{i,j\in\{\text{Loci}\}\\ j>i+2}} U_{sc}(\vec{r}_{i,j}), \end{aligned} \tag{1}$$

U_{FENE} (Finite Extensible Nonlinear Elastic potential) is the bonding term applied between two consecutive monomers, connecting a sequence of beads with nonlinear springs.[13] K_b is the spring constant and R_0 is the equilibrium bond length.

$$U_{\text{FENE}}(\vec{r}_{i,j}) = \begin{cases} -\frac{1}{2} K_b R_0^2 \ln\left[1 - \left(\frac{r_{i,j}}{R_0}\right)\right] & r_{i,j} \le R_0 \\ 0 & r_{i,j} > R_0 \end{cases} \tag{2}$$

Additionally, the hard-core repulsive potential $U_{hc}(\vec{r}_{i,j})$ is included to avoid overlap between bonded monomers:

$$U_{hc}(\vec{r}_{i,j}) = \begin{cases} 4\epsilon\left[\left(\frac{\sigma}{r_{i,j}}\right)^{12} - \left(\frac{\sigma}{r_{i,j}}\right)^{6} + \frac{1}{4}\right] & r_{i,j} \le \sigma 2^{\frac{1}{6}} \\ 0 & r_{i,j} > \sigma 2^{\frac{1}{6}} \end{cases} \tag{3}$$

A three-body interaction is included to all connected three consecutive monomers to consider a nonzero bending stiffness.[14] This angular potential that regulates the chain flexibility is given by:

$$U_{\text{Angle}}(\theta_i) = K_a[1 - \cos\theta_i - \theta_0], \tag{4}$$

where θ_i is the angle defined by two vectors $\vec{r}_{i,i+1}$ and $\vec{r}_{i,i-1}$.

All non-bonded pair interactions are described by a soft-core repulsive potential with the following form:

$$U_{sc}(\vec{r}_{i,j}) = \begin{cases} \frac{1}{2}E_{\text{cut}}\left[1 + \tanh\left(\frac{2U_{LJ}(\vec{r}_{i,j})}{E_{\text{cut}}} - 1\right)\right] & r_{i,j} \leq r_0 \\ U_{LJ}(\vec{r}_{i,j}) & r_0 \leq r_{i,j} \leq \sigma 2^{\frac{1}{6}} \\ 0 & r_{i,j} > \sigma 2^{\frac{1}{6}} \end{cases} \tag{5}$$

The expression U_{LJ} corresponds to the Lennard-Jones potential $U_{LJ}(\vec{r}_{i,j}) = 4\epsilon\left[\left(\frac{\sigma}{r_{i,j}}\right)^{12} - \left(\frac{\sigma}{r_{i,j}}\right)^{6} + \frac{1}{4}\right]$ capped off at a finite distance, thus allowing for chain crossing at finite energetic cost (topoisomerases activity). r_0 is chosen as the distance at which $U_{LJ}(\vec{r}_{i,j}) = \frac{1}{2}E_{cut}$. The model parameters are included in reduced units as:

$$K_a = 2\epsilon \quad K_b = \frac{30\epsilon}{\sigma^2} \quad E_{cut} = 4\epsilon \quad \epsilon = KT \quad R_0 = 1.5\sigma \quad \sigma = 1 \quad \theta_0 = \pi$$

Centromere and Telomeres Position Restraints

Centromeric and telomeric regions are anchored to the nucleus wall using a flat-bottomed potential U_{rfb}. This potential is employed to restrain the loci within a simulation volume. If a locus moves outside the chosen region, a harmonic force moves the bead to the flat-bottomed region. On the other hand, there is no force acting on the centromeric and telomeric locus within the flat-bottomed part of the potential. The flat-bottomed potential is described as follows:

$$U_{\text{rfb}}(\vec{r}_i) = -\frac{1}{2}k_{rfb}(r_i - R_{f0})^2\Theta(r_i - R_{f0}) \tag{6}$$

where $R_{f0} = 2.5\sigma$ is the reference distance of the flat bottom potential, r_i is the locus's distance from the reference, $k_{rfb} = \frac{0.2\epsilon}{\sigma^2}$ the force constant, and Θ is the Heaviside step function. The position restraints applied to centromeres and telomeres to maintain the polarized architecture can be associated with the force/tension of their interaction with the nucleus membrane. Increasing/decreasing the centromere-telomeres spatial distance would mimic the effect of the nucleus deformation by expansion and compression. Supplementary Table 2 shows the set of beads that belong to a certain region for each chromosome.

Supplementary Table 2: Set of beads that belong to a certain region (centromeric or telomeric) for each chromosome. C label the centromere, T1 and T2 labels the two telomeres.

	C	T1	T2
1P/1M	1-15	1510-1550	3095-3109
2P/2M	1-15	2250-2350	4730-4744
3P/3M	1-15	1975-2000	4084-4098

Crosslinking Probability Function

One challenge for modeling chromosomes based on Hi-C maps is the relationship between the crosslink probability in the experimental and the geometric distance of a loci pair. MiChroM modeling assumes that the probability of a loci pair i and j to form contact in 3D space decreases as a function of their distance $r_{i,j}$. In this sense, $f(r_{i,j})$ is adopted as a sigmoid function (switch function) designed to consider a high probability of crosslinking for short distances while long geometric distance should have a low probability of contact:

$$f(r_{i,j}) = \frac{1}{2}\left(1 + \tanh\left[\mu(r_c - r_{i,j})\right]\right) \tag{7}$$

where μ and r_c values are determined based on the experimental Hi-C maps. The adjustment of the parameters considers two criteria: i) the function $f(r_{i,j})$ is calibrated to return 1 when two beads are in contact (distance between the center of two beads is equal to 1, in

reduced units σ), *i.e.*, $f(1) = 1$. *ii*) $f(r_{i,j})$ is also tuned to correctly return the experimental probability for the nearest neighbors where the maximum distance between the next nearest neighbors is 2 in reduced units σ. $f(r_{i,j})$ should decrease monotonically with the distance and the minimum value of the experimental probabilities must match with the next nearest neighbor maximum distance, *i.e.*, $f(2) = \min\{P^{\text{exp}}_{i,i+2}\}$. The parameters adjusted for the Hi-C maps of *Aedes aegypti* are $\mu = 3.48$ and $r_c = 1.76$.

Orientation Order Parameter - O_{OP}

The orientation order parameter O_{OP} is defined as the correlation between two unit vectors connecting beads $[i,\, i+4]$ and $[j,\, j+4]$ and it is described as follows:

$$O_{OP}(i-j) = \langle \overrightarrow{r_{i,i+4}} \cdot \overrightarrow{r_{j,j+4}} \rangle \tag{8}$$

where $\overrightarrow{r_{i,i+4}}$ is the unit vector that points from the i-th to the $i+4$-th locus. The brackets $\langle\cdot\rangle$ is the average over an ensemble of the chromosome structures. The analysis code of the order parameter O_{OP} is now implemented into cndbTools function as a part of the Open-MiChroM software package for chromatin dynamics simulations (`https://open-michrom.readthedocs.io`).[4,5]

Energy Function Parameters from the Optimization

The *Aedes aegypti* genome was modeled employing the MiChroM energy function on chromosome 1 obtained from the Hi-C map[8,9] with a resolution of 100 kb per bead. The potential parameters were optimized for the type-to-type interaction to accommodate for the AB phase separation and for the ideal chromosome to consider the motor activity. Supplementary Table 3 presents the values of the chromatin types interactions for the mosquito genome. Given that the *Aedes aegypti* genome was modeled at 100 kb, the strength of the parameters for the chromatin types is similar to the obtained for humans.[2,4] Supplementary Fig. 7A presents

the optimization energy function for the ideal chromosome fitted on the data point obtained during the training. Supplementary Fig. 7B shows the scatter plot using the points extracted from the simulated and experimental Hi-C maps.

Supplementary Table 3: Type-to-type interaction values for the *Aedes aegypti* genome in units of KT.

	A	B
A	-0.252	-0.273
B	-0.273	-0.339

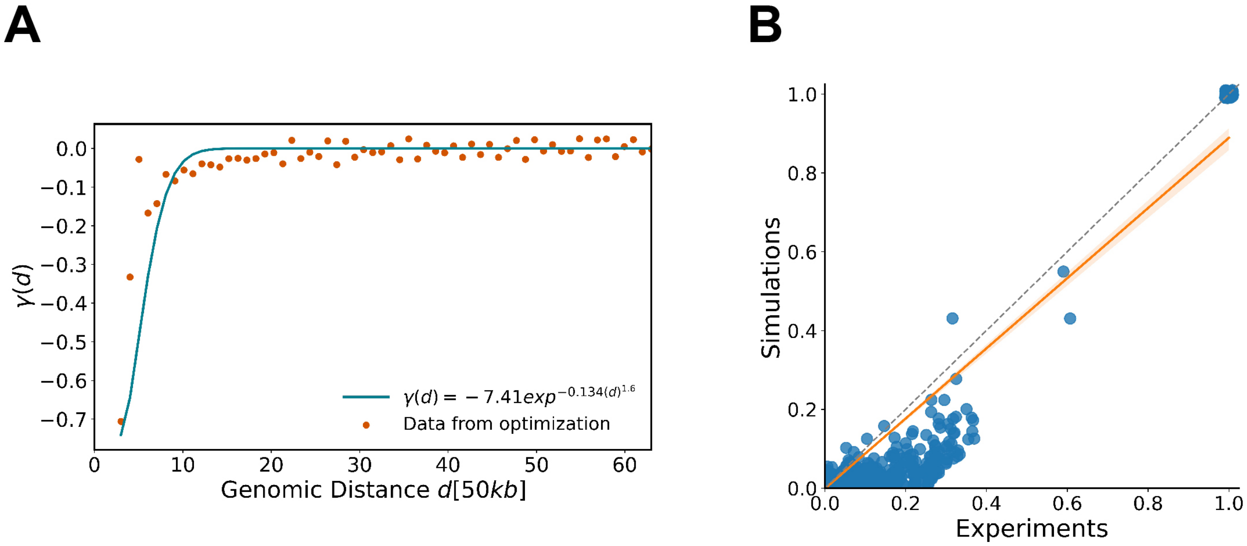

Supplementary Fig. 7: A - Ideal chromosome function fitted on data points obtained from the optimization employing a stretched exponential function. B - Scatter plot comparing the points extracted from the simulated and experimental Hi-C maps. The linear fit parameters are slope = 0.89, intercept = -0.001523, r_value=0.9574, std_err = 0.001221. For better rendering, only 5% of the points are presented on the plot.

Supplementary References

(1) Zhang, B.; Wolynes, P. G. Topology, Structures, and Energy Landscapes of Human Chromosomes. *Proceedings of the National Academy of Sciences* **2015**, *112*, 6062–6067.

(2) Pierro, M. D.; Zhang, B.; Aiden, E. L.; Wolynes, P. G.; Onuchic, J. N. Transferable Model for Chromosome Architecture. *Proceedings of the National Academy of Sciences* **2016**, *113*, 12168–12173.

(3) Contessoto, V. G.; Cheng, R. R.; Hajitaheri, A.; Dodero-Rojas, E.; Mello, M. F.; Lieberman-Aiden, E.; Wolynes, P. G.; Di Pierro, M.; Onuchic, J. N. The Nucleome Data Bank: Web-Based Resources to Simulate and Analyze the Three-Dimensional Genome. *Nucleic Acids Research* **2021**, *49*, D172–D182.

(4) Oliveira Junior, A. B.; Contessoto, V. G.; Mello, M. F.; Onuchic, J. N. A Scalable Computational Approach for Simulating Complexes of Multiple Chromosomes. *Journal of Molecular Biology* **2021**, *433*, 166700.

(5) Oliveira Junior, A. B.; Estrada, C. P.; Aiden, E. L.; Contessoto, V. G.; Onuchic, J. N. Chromosome Modeling on Downsampled Hi-C Maps Enhances the Compartmentalization Signal. *The Journal of Physical Chemistry B* **2021**, *125*, 8757–8767.

(6) Hoencamp, C.; Dudchenko, O.; Elbatsh, A. M.; Brahmachari, S.; Raaijmakers, J. A.; van Schaik, T.; Cacciatore, Á. S.; Contessoto, V. G.; van Heesbeen, R. G.; van den Broek, B., et al. 3D genomics across the tree of life reveals condensin II as a determinant of architecture type. *Science* **2021**, *372*, 984–989.

(7) Rao, S. S. P.; Huntley, M. H.; Durand, N. C.; Stamenova, E. K.; Bochkov, I. D.; Robinson, J. T.; Sanborn, A. L.; Machol, I.; Omer, A. D.; Lander, E. S.; Aiden, E. L. A 3D Map of the Human Genome at Kilobase Resolution Reveals Principles of Chromatin Looping. *Cell* **2014**, *159*, 1665–1680.

(8) Dudchenko, O.; Batra, S. S.; Omer, A. D.; Nyquist, S. K.; Hoeger, M.; Durand, N. C.; Shamim, M. S.; Machol, I.; Lander, E. S.; Aiden, A. P., et al. De novo assembly of the Aedes aegypti genome using Hi-C yields chromosome-length scaffolds. *Science* **2017**, *356*, 92–95.

(9) Matthews, B. J.; Dudchenko, O.; Kingan, S. B.; Koren, S.; Antoshechkin, I.; Crawford, J. E.; Glassford, W. J.; Herre, M.; Redmond, S. N.; Rose, N. H., et al. Improved reference genome of Aedes aegypti informs arbovirus vector control. *Nature* **2018**, *563*, 501–507.

(10) Knight, P. A.; Ruiz, D. A Fast Algorithm for Matrix Balancing. *IMA Journal of Numerical Analysis* **2013**, *33*, 1029–1047.

(11) Durand, N. C.; Shamim, M. S.; Machol, I.; Rao, S. S. P.; Huntley, M. H.; Lander, E. S.; Aiden, E. L. Juicer Provides a One-Click System for Analyzing Loop-Resolution Hi-C Experiments. *Cell Systems* **2016**, *3*, 95–98.

(12) Hodges, J. L. The significance probability of the Smirnov two-sample test. *Arkiv för Matematik* **1958**, *3*, 469–486.

(13) Kremer, K.; Grest, G. S. Dynamics of Entangled Linear Polymer Melts: A Molecular-dynamics Simulation. *The Journal of Chemical Physics* **1990**, *92*, 5057–5086.

(14) Auhl, R.; Everaers, R.; Grest, G. S.; Kremer, K.; Plimpton, S. J. Equilibration of Long Chain Polymer Melts in Computer Simulations. *The Journal of Chemical Physics* **2003**, *119*, 12718–12728.
