# Supplementary material for: Interphase chromosomes of the Aedes aegypti mosquito are liquid crystalline and can sense mechanical cues
Source: Nat Commun. 2023 Jan 19;14:326. doi: 10.1038/s41467-023-35909-2 (PMC9852290; doi:10.1038/s41467-023-35909-2)
Supplement: Supplementary file 3 — Reporting Summary [file 41467_2023_35909_MOESM3_ESM.pdf]

nature portfolio

Corresponding author(s): Vinicius Contessoto, Michele Di Pierro

Last updated by author(s): Dec 6, 2022

Reporting Summary

Nature Portfolio wishes to improve the reproducibility of the work that we publish. This form provides structure for consistency and transparency in reporting. For further information on Nature Portfolio policies, see our Editorial Policies and the Editorial Policy Checklist.

Statistics

For all statistical analyses, confirm that the following items are present in the figure legend, table legend, main text, or Methods section.

n/a	Confirmed	
☒	☐	The exact sample size (*n*) for each experimental group/condition, given as a discrete number and unit of measurement
☒	☐	A statement on whether measurements were taken from distinct samples or whether the same sample was measured repeatedly
☐	☒	The statistical test(s) used AND whether they are one- or two-sided<br>*Only common tests should be described solely by name; describe more complex techniques in the Methods section.*
☒	☐	A description of all covariates tested
☒	☐	A description of any assumptions or corrections, such as tests of normality and adjustment for multiple comparisons
☒	☐	A full description of the statistical parameters including central tendency (e.g. means) or other basic estimates (e.g. regression coefficient) AND variation (e.g. standard deviation) or associated estimates of uncertainty (e.g. confidence intervals)
☒	☐	For null hypothesis testing, the test statistic (e.g. *F*, *t*, *r*) with confidence intervals, effect sizes, degrees of freedom and *P* value noted<br>*Give P values as exact values whenever suitable.*
☒	☐	For Bayesian analysis, information on the choice of priors and Markov chain Monte Carlo settings
☒	☐	For hierarchical and complex designs, identification of the appropriate level for tests and full reporting of outcomes
☐	☒	Estimates of effect sizes (e.g. Cohen's *d*, Pearson's *r*), indicating how they were calculated

Our web collection on statistics for biologists contains articles on many of the points above.

Software and code

Policy information about availability of computer code

Data collection	Simulations performed using https://www.gromacs.org - GROMACS package version 2016.3
Data analysis	Analysis were performed using Python 3. Chromosomal 3D structures were represented using Chimera alpha channel and VMD version 1.9.4. OpenMiChroM version 1.0.5 software with CNDBtools were used for data analysis (https://github.com/junioreif/OpenMiChroM).

For manuscripts utilizing custom algorithms or software that are central to the research but not yet described in published literature, software must be made available to editors and reviewers. We strongly encourage code deposition in a community repository (e.g. GitHub). See the Nature Portfolio guidelines for submitting code & software for further information.

Data

Policy information about availability of data

All manuscripts must include a data availability statement. This statement should provide the following information, where applicable:

- Accession codes, unique identifiers, or web links for publicly available datasets
- A description of any restrictions on data availability
- For clinical datasets or third party data, please ensure that the statement adheres to our policy

We included the Data availability and the Code availability statements in the main text.
Data availability: The authors declare that the data supporting this manuscript's findings are present in the main text or the Supplementary Information. All the publicly available datasets used in the study is followed by the accessible links/accession-codes. Additional relevant data are available from the corresponding

authors upon reasonable request.
\section*{Code availability}The simulation code using OpenMiChroM is available at https://github.com/junioreif/OpenMiChroM. The code documentation with details on how to perform the chromatin dynamics simulations is available at https://open-michrom.readthedocs.io. The GROMACS molecular dynamics package is available at the Nucleome Data Bank (ndb.rice.edu). Analysis codes are now part of the CNDBtools implemented into the OpenMiChroM platform.

Human research participants

Policy information about studies involving human research participants and Sex and Gender in Research.

Reporting on sex and gender	n/a
Population characteristics	n/a
Recruitment	n/a
Ethics oversight	n/a

Note that full information on the approval of the study protocol must also be provided in the manuscript.

Field-specific reporting

Please select the one below that is the best fit for your research. If you are not sure, read the appropriate sections before making your selection.

[x] Life sciences [ ] Behavioural & social sciences [ ] Ecological, evolutionary & environmental sciences

For a reference copy of the document with all sections, see nature.com/documents/nr-reporting-summary-flat.pdf

Life sciences study design

All studies must disclose on these points even when the disclosure is negative.

Sample size	We used ATAC-seq data with reference number PRJNA418406 (https://www.ncbi.nlm.nih.gov/bioproject/?term=PRJNA418406) and SRX code SRX3580386 (https://www.ncbi.nlm.nih.gov/sra/SRX3580386[accn]) to explorethe 3D spatial location of High and Low intensity signal. High-intensity ATAC-seq signals are selected on values belonging to the 95th percentile and higher. On the other hand, Low-intensity signals are based on the 5th percentile and lower.
Data exclusions	no data was excluded
Replication	We selected high and Low signals based on the 5th and 95th percentile. No replica from the distribution is necessary.
Randomization	We selected high and Low signals based on the 5th and 95th percentile. No randomization in the selection and comparison is necessary.
Blinding	We selected high and Low signals based on the 5th and 95th percentile. No blinding is necessary to select or analyze this data.

Reporting for specific materials, systems and methods

We require information from authors about some types of materials, experimental systems and methods used in many studies. Here, indicate whether each material, system or method listed is relevant to your study. If you are not sure if a list item applies to your research, read the appropriate section before selecting a response.

Materials & experimental systems

n/a	Involved in the study	
[x]	[ ]	Antibodies
[x]	[ ]	Eukaryotic cell lines
[x]	[ ]	Palaeontology and archaeology
[x]	[ ]	Animals and other organisms
[x]	[ ]	Clinical data
[x]	[ ]	Dual use research of concern

Methods

n/a	Involved in the study	
[x]	[ ]	ChIP-seq
[x]	[ ]	Flow cytometry
[x]	[ ]	MRI-based neuroimaging
